# Phytochemistry, health benefits, and food applications of sea buckthorn (*Hippophae rhamnoides* L.): A comprehensive review

**DOI:** 10.3389/fnut.2022.1036295

**Published:** 2022-12-06

**Authors:** Zhen Wang, Fenglan Zhao, Panpan Wei, Xiaoyun Chai, Guige Hou, Qingguo Meng

**Affiliations:** ^1^Key Laboratory of Molecular Pharmacology and Drug Evaluation (Yantai University), Ministry of Education, Collaborative Innovation Center of Advanced Drug Delivery System and Biotech Drugs in Universities of Shandong, School of Pharmacy, Yantai University, Yantai, China; ^2^Department of Organic Chemistry, School of Pharmacy, Naval Medical University, Shanghai, China; ^3^School of Pharmacy, Binzhou Medical University, Yantai, China

**Keywords:** sea buckthorn, phytochemistry, nutrients, health benefits, food applications

## Abstract

Sea buckthorn (*Hippophae rhamnoides* L.), an ancient miraculous plant, is of great interest because of its tenacity, richness in nutritional active substances, and biological activity. Sea buckthorn is a deciduous shrub or tree of the genus *Hippophae* in the family *Elaeagnaceae*. It is a pioneer tree species for soil improvement, wind and sand control, and soil and water conservation. Sea buckthorn contains many nutritional active components, such as vitamins, carotenoids, polyphenols, fatty acids, and phytosterols. Moreover, sea buckthorn has many health benefits, such as antioxidant, anticancer, anti-hyperlipidemic, anti-obesity, anti-inflammatory, antimicrobial, antiviral, dermatological, neuroprotective, and hepatoprotective activities. Sea buckthorn not only has great medicinal and therapeutic potential, but also is a promising economic plant. The potential of sea buckthorn in the human food industry has attracted the research interest of researchers and producers. The present review mainly summarizes the phytochemistry, nutrients, health benefits, and food applications of sea buckthorn. Overall, sea buckthorn is a dietary source of bioactive ingredients with the potential to be developed into functional foods or dietary supplements for the prevention and treatment of certain chronic diseases, which deserves further research.

## Introduction

Sea buckthorn (*Hippophae rhamnoides* L.) is a deciduous shrub or tree that is also known as Siberian pineapple, sand thorn, sea berry, and sallow thorn (1). *Hippophae* L. originated in the Hengduan Mountains and East Himalayas area and is widely distributed in the temperate regions of Eurasia (2). Every part of this plant (fruits, leaves, stems, branches, roots, and thorns) has been traditionally used in medicine, nutritional supplement, soil and moisture conservation, and the establishment of wildlife habitats. Therefore, sea buckthorn is popularly known as “Wonder Plant,” “Golden Bush,” or “Gold Mine” (3).

Since the 1940s, Russian scientists have researched the bioactive substances in the berries, leaves and bark of sea buckthorn, leading to the development of sea buckthorn foods and radiation protection creams for Russian cosmonauts (4). Sea buckthorn contains nearly 200 nutritional and bioactive compounds and is known as a “natural vitamin treasure house” and a “source of nutrition and health care” (5, 6). Sea buckthorn is therefore widely used by the food industry in the preparation of breads, yogurts, jams, beverages, teas and other products (7–9). The medicinal value of sea buckthorn has been recorded in the Tibetan medical classic “Somaratsa,” dating back to as early as the first half of the eighth century (10). Sea buckthorn has been extensively exploited in the folklore treatment of slow digestion, stomach malfunctioning, cardiovascular problems, liver injury, skin diseases, and ulcers (11). In recent years, there have been numerous reports on the pharmacological activities of sea buckthorn, including its anticancer, anti-inflammatory, antimicrobial and antiviral activities, and its ability to act in cardiovascular protection (12–16). There is no doubt that sea buckthorn has great medicinal and therapeutic potential, which may be attributed to the fact that sea buckthorn contains several vitamins, carotenoids, polyphenols, and fatty acids (17–20).

Sea buckthorn is a plant of ecological and economic importance. To promote the role of sea buckthorn in environmental protection, economic development, and human health, the International Sea Buckthorn Association (ISA) was established in 1999 by China, India, Canada, and other countries. In recent years, more countries have become aware of the therapeutic potential of sea buckthorn, and many countries are beginning to recognize and develop a sea buckthorn industry. According to statistics, as of December 2020, sea buckthorn had been distributed to 52 countries around the world, with a total area of 2.33 million hm^2^. Among this distribution, about 2.1 million hm^2^ is found within China and the rest is in other countries (21). With the increase of sea buckthorn planting and production, more attention should be paid to the exploitation and utilization of sea buckthorn. Thus, the present review aims to provide a comprehensive overview of the phytochemistry, nutritional and bioactive compounds, health benefits and food applications of sea buckthorn for reference by industrial manufacturers and researchers.

## Botanical description

### Morphology

**FIGURE 1:**
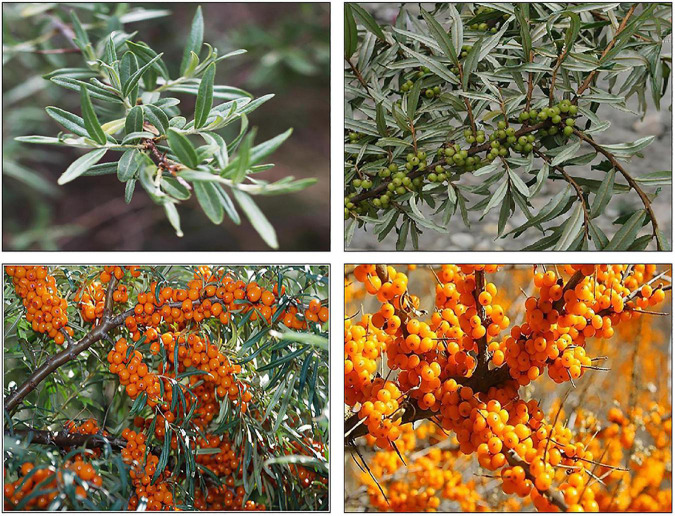
Sea buckthorn in different periods.

Sea buckthorn is a deciduous tree or shrub of the *Elaeaceae* family and *Hippophae* L. genus (Figure 1). It is generally 1–8 m high, with some plants growing up to 18 m tall. The leaves are lanceolate or linear, usually 3–8 cm long and less than 7 mm wide. The upper surface of the leaves is dark gray, and the lower surface is distinct silver-gray (22). The fruits are spherical or oblate with a diameter of 5–8 mm. There are usually several fruits stuck together. The fruit is orange-yellow or brownish-red in color and has a ruffled surface. The pulp is oily and soft in texture. The seeds of sea buckthorn are about 4 mm long, 2 mm wide, and obliquely ovate. The seeds are brown and shiny, with a longitudinal groove in the middle. The seed coat is hard, and the seed kernel is creamy white (Figure 1) (23).

### Taxonomy

On the basis of the analysis of morphological variation, Arne Rousi classified *Hippophae* L. (2*n* = 24) into three species, *H. rhamnoides* L., *H. salicifolia* D. Don, and *H. tibetana* Schlecht. *H. rhamnoides* is divided into nine subspecies, which include the ssp. *carpatica* Rousi, ssp. *caucasica* Rousi, ssp. *gyantsensis* Rousi, ssp. *mongolica* Rousi, ssp. *sinensis* Rousi, ssp. *turkestanica* Rousi, ssp. *yunnanensis* Rousi, ssp. *rhamnoides*, and ssp. *fluviatilis* van Soest (24). Liu and He described a fourth species, *H. neurocarpa.* Liu and He, found on the Qinghai-Tibet Plateau of China (25). However, taxonomists still disagree on the precise classification of the genus *Hippophae*. Chinese scientist Hu has updated and improved the classification system and revised *Hippophae* L. into 6 species and 17 subspecies (Table 1) (26).

**TABLE 1:** Classification of *Hippophae* Linn.

Genus	Section	Species	Subspecies
*Hippophae* L.	*Hippophae*	*Hippophae salicifolia*	
		*Hippophae rhamnoides*	ssp. *sinensis*
			ssp. *yunnanensis*
			ssp. *turkestanica*
			ssp. *mongolica*
			ssp. *caucasica*
			ssp. *carpatica*
			ssp. *rhamnoides*
			ssp. *fluviatilis*
	*Gyantsenses*	*Hippophae goniocarpa*	ssp. *litangensis*
			ssp. *goniocarpa*
		*Hippophae gyantsensis*	ssp. *litangensis*
			ssp. *gyantsensis*
		*Hippophae neurocarpa*	ssp. *stellatopilosa*
			ssp. *neurocarpa*
		*Hippophae tibetana*	ssp. *tibetana*

## Nutrients and bioactive compounds

Sea buckthorn contains nearly 200 nutrients and bioactive components (5). Many of the components are well known for their health benefits. Vitamin C is a very important nutrient in sea buckthorn. Carotenoids and polyphenolic compounds, especially phenolic acids and flavonoids, are the main bioactive and antioxidant components of sea buckthorn (27). The fatty acids, phytosterols, organic acids, amino acids, and minerals contained in sea buckthorn also play an important role. The nutrients and bioactive composition content of sea buckthorn influence its health value (28). The nutritional and bioactive composition of sea buckthorn fruit varies considerably depending on genetic variation, the part analyzed, climatic, and growth conditions, year of harvest, degree of maturity, storage conditions, harvest time, and processing and analytic methods (29). Tables 2, 3 show the main nutrients and bioactive components in sea buckthorn fruits, respectively.

**TABLE 2:** Nutrients in sea buckthorn fruits.

Class	Compounds	Content	Analytical method	Specie/varieties	Location	References
**Vitamin** (mg/100 g)	Vitamin C	275	HPLC	–	Ladakh	(17)
	Vitamin E	3.54				
	Vitamin B_12_	5.4				
	Riboflavin	1.45				
	Vitamin B_6_	1.12				
	Niacin	68.4				
	Vitamin A	432.4 IU/100 g				
	Pantothenic acid	0.85 mcg/100 g				
**Mineral**	Phosphorus (P)	491	HPLC	Wild	Iran	(30)
(mg/kg DW)	Potassium (K)	1,674				
	Calcium (Ca)	1,290				
	Magnesium (Mg)	990				
	Iron (Fe)	291				
	Zinc (Zn)	29.77				
	Manganese (Mn)	108.37				
	Copper (Cu)	17.87				
(mg/kg DM)	Boron (B)	13.61–16.3	ICP-AES	Wild ssp. *rhamnoides* and Mažeikiai	Lithuania	(31)
	Nickel (Ni)	0.41–0.49	GFAAS			
	Cadmium (Cd)	0.023–0.045				
	Lead (Pb)	0.034–0.038				
**Organic acid** (mg/100 g)	L-malic acid	26.45	HPLC	Botanicheskaya	Japan	(38)
	D-malic acid	22.90				
	Succinic acid	6.96				
	Citric acid	2.01				
	Tartaric acid	5.39				
	Quininic acid	16.00				
	Pyruvic acid	0.32				
	Acetic acid	0.08				
	Formic acid	1.94				
	Oxalic acid	10–20	HPLC, UPLC	Aromatnaja, Botaniczeskaja-Lubitelskaja, Józef, Luczistaja, Moskwiczka, and Podarok Sadu	Poland	(34)
	Isocitric acid	20–21				
**Amino acid** (mg/100 g)	Aspartic acid	240–560	HPLC	Shenqiuhong, Wucifeng, Xinjiyihao, Xinjierhao	China	(37)
	Serine	11–80				
	Glutamic acid	260–360				
	Glycine	60–80				
	Alanine	56–92				
	Cysteine	20–41				
	Tyrosine	38–51				
	Histidine	56–62				
	Arginine	130–180				
	Proline	64–130				
	Threonine	47–63				
	Valine	74–92				
	Methionine	11–20				
	Isoleucine	52–80				
	Leucine	90–130				
	Phenylalanine	60–82				
	Lysine	78–93				
	Total	1,460–2,190				

“–” indicates that the value is not available. DW, dry weight basis; DM, dry matter basis.

**TABLE 3:** Main bioactive components in sea buckthorn fruits.

Class	Compounds	Content	Analytical method	Specie/varieties	Location	References
**Carotenoids** (mg/100 g DW)	Lutein	1.4–2.1	HPLC-PAD	ssp. *Carpatica* (Victoria, Tiberiu, Sf. Gheorghe, Serpenta, Serbanesti 4, and Ovidiu)	Carpathians	(18)
	Zeaxanthin	1.8–2.5				
	β-Cryptoxanthin	1.3–1.6				
	δ-Carotene	1.4–1.9				
	α-Carotene	0.9–1.6				
	γ-Carotene	1.6–1.8				
	*Cis* β-Carotene	1.3–2.1				
	β-Carotene	1.9–7.5				
(mg/100 g)	Lycopene	13–20	UV/VIS	–	Iran	(29)
(mg/100 g DM)	Xanthophylls	37.76–80.73	UPLC	Aromatnaja, Botaniczeskaja-Lubitelskaja, Józef, Luczistaja, Moskwiczka, and Podarok Sadu	Poland	(34)
	Total carotenoids	53–97		ssp. *Carpatica*	Carpathians	(18)
**Polyphenols** (mg/kg DM)	Phenolic acid	51.8–89.4	UPLC	Aromatnaja, Botaniczeskaja-Lubitelskaja, Józef, Luczistaja, Moskwiczka, and Podarok Sadu	Poland	(34)
		3,570–4,439	GC-MS	Nadba, ltycka, Nevlejena, Otradnaja, Podarok, Sadu, Trofimowskaja, and Hybrid 29–88	Poland and Byelorussia	(46)
	Ferulic acid	5.1–17.8				
	Gallic acid	1.0–4.6				
	Vanillic acid	1.4–8.4				
	Caffeic acid	0.9–6.7				
	Protocatechuic acid	0.7–4.3				
	*m*-Coumaric	0.3–6.1				
	*o*-Coumaric	2.2–13.3				
	*p*-Coumaric acid	1.4–9.8				
	Quinic	3.5–193.9				
	Cinnamic	0.8–803.9				
	2,5-Dihydroxybenzoic	0.1–3.1				
	3,4-Dihydroxycinnamic	5.9–27.3				
	Pyrocatechuic	0.2–32.1				
	Salicylic	21–47.5				
	*p*-Hydroxyphenyl-lactic	5.3–24.7				
	Veratric	3.3–63				
	Hydroxycaffeic	9.1–58.5				
	Syringic	2.5–12.8				
(mg/kg FW)	flavonoid	381–616	RP-HPLC	Avgustinka, Botanièeskaja, Botanièeskaja Liubitelskoje, Hibrid perèika, Julia, Nivelena-1, Nivelena-2, Otradnaja, Padarok sadu, Trofimovskaja, Vorobjevskaja	Lithuania	(49)
	quercetin-3-O-rutinoside	99–280				
	isorhamnetin-3-O-rutinoside	102–229				
	isorhamnetin-3-O-glucoside	83–195				
(mg/mL)	Catechin	8.51	HPLC	Botanicheskaya	Japan	(38)
	Rutin	9.45				
	Quercetin	5.16				
(mg/100 g DW)	Isorhamnetin	10.3–15.1	RP-HPLC	ssp. *sinensis*, ssp. *yunnanensis*, ssp. *mongolica*, ssp. *turkestanica*	China	(48)
	Kaempferol	1.02–1.5				
	Quercetin-3-*O*-rutinoside	23–44.6				
	Quercetin-3-*O*-glucoside	31.2–49.5				
	Isorhamnetin-3-*O*-rutinoside	38.7–84.0				
	Isorhamnetin-3-*O*-glucoside	7.61–26.0				
	Kaempferol-3-*O*-sophoroside-7-*O*-rhamnoside	34.1–61.6				
	Isorhamnetin-3-*O*-sophoroside-7-*O*-rhamnoside	15.2–74.6				
	Isorhamnetin-3-*O*-glucoside-7-*O*-rhamnoside	112–187				
**Fatty acids** (g/kg)	Myristic (C14:0)	2–2.6	GC	wild ssp. *rhamnoides* and Mažeikiai	Lithuania	(31)
	Pentadecanoic (C15:0)	0.93–2.23				
	Palmitic (C16:0)	223.2–227.2				
	Margaric (C17:0)	0.56–0.86				
	Stearic (C18:0)	13.25–17.86				
	Arachidic (C20:0)	2.81–3.72				
	Henicosanoic (C21:0)	0.73–1.54				
	Behenic (C22:0)	1.09–2.28				
	Lignoceric (C24:0)	0.60–0.93				
	Myristoleic (C14:1)	0.31–1.53				
	Pentadecenoate (C15:1)	0.00–0.60				
	Palmitoleic (C16:1 n-7)	134.6–185.0				
	Hexadecenoic (C16:1 n-9)	0.80–0.91				
	Margaroleic (C17:1)	0.44–0.71				
	Oleic (C18:1 n-9)	255.5–264.1				
	*cis*-Vaccenic (C18:1 n-7)	56.52–65.61				
	Eicosenoic (C20:1 n-9)	2.50–2.73				
	Erucic (C22:1 n-9)	1.06–1.07				
	Nervonic (C24:1 n-9)	1.00–1.51				
	Linoleic (C18:2 n-6)	127.0–163.5				
	γ-Linolenic (C18:3 n-6)	0.30–0.60				
	α-Linolenic (C18:3 n-3)	100.3–109.8				
	Docosatetraenoic (C22:4 n-6)	3.90–4.41				
	Docosapentaensyre (C22:5 n-3)	2.53–2.60				
**Phytosterols** (μg/100 mL)	Squalene	885.71–2714.37	GC	ssp. *Mongolica* (Aromatnaja, Avgustinka, Botaniczeskaja, Botaniczeskaja Ljubitelskaja, Luczistaja, Moskwiczanka, Podarok Sadu, and Porozrachnaja)	Poland	(28)
	Kampesterol	44.37–201.32				
	Stigmasterol	24.08–68.22				
	β-Sitosterol	2036.14–6145.58				
	Sitostanol	96.50–254.67				
	Δ^5^-Avenasterol	114.93–377.56				
	α-Amyrin	110.48–314.52				
	Cycloarteno	293.49–474.38				
	Δ^7^-Avenasterol	80.80–194.97				
	28-Methylobtusifoliol	70.88–251.85				
	24-Methylenecycloartanol	1454.21–4048.89				
	Erythrodiol	284.02–818.75				
	Citrostadienol	212.97–663.22				
	Friedelan-3-ol	232.89–737.28				
	Total	6168.24–13378.22				

FW, fresh weight basis; DW, dry weight basis; DM, dry matter basis.

### Nutrients

#### Vitamins and minerals

The quality of sea buckthorn fruit is often based on its nutritional value (29). Known as a “natural treasure trove of vitamins,” sea buckthorn is undoubtedly rich in vitamins (6). The vitamin C content of sea buckthorn fruits ranges from 52.86 to 896 mg/100 g (28, 29). It has been showed that the vitamin C content of 100 g of sea buckthorn berries (275 mg) is much higher than the equivalent quantity of mango (27.7 mg), apricot (10 mg), banana (8.7 mg), orange (50 mg), and peach (6.6 mg) (17). In addition, sea buckthorn berries contain vitamin A, vitamin E, riboflavin, niacin, pantothenic acid, vitamin B_6_, and vitamin B_12_. Mineral elements are involved in the formation of human tissues and the maintenance of normal physiological functions. Sea buckthorn berries contain many minerals, e.g., phosphorus, iron, magnesium, boron, calcium, aluminum, potassium and others (30, 31). Significant differences in the mineral content of sea buckthorn fruits have been reported at its different stages of maturity. The highest content of calcium, magnesium and phosphorus was found in ripe sea buckthorn fruits with 68.28, 145.67, and 457.7 mg/kg, respectively (32).

#### Carbohydrates

As the main component of dry matter, carbohydrates play numerous essential roles in living organisms. Monosaccharides are the main source of energy for human metabolism with polysaccharides acting as structural components and the main storage form of energy (33). Sugar content determines the sweetness of the juice. It has been reported that sea buckthorn fruits contain 1.34–2.87 g/100 g FW of sugar. The sugar with the highest content is glucose, accounting for 86.58–92.68% of the total sugar content (34). A study on the sugar composition of three German sea buckthorn varieties reported that the contents of glucose, fructose, and mannitol are 11.95–15.26 mg/mL, 1.75–6.75 mg/mL, and 1.32–6.21 mg/mL, respectively. The sugar content varies among varieties (35).

#### Organic acids and amino acids

Sea buckthorn fruit contains several organic acids and their derivatives. These organic acid derivatives can promote bone differentiation and contribute to the differentiation of mesenchymal stem cells into osteoblasts (36). Different species of sea buckthorn have different types and concentrations of organic acids. For example, subspecies of Russian sea buckthorn exhibit relatively low total acidity, with organic acid concentrations of 2.1–3.2 g/100 mL. Finnish genotypes were in the middle, ranging from 4.2 to 6.5 g/100 mL, whereas Chinese genotypes showed the highest organic acid concentration, with values between 3.5 and 9.1 g/100 mL (37). It has been reported that sea buckthorn juice contains nine organic acids, namely quinic acid, L-malic acid, D-malic acid, succinic acid, pyruvic acid, tartaric acid, acetic acid, formic acid, and citric acid (38). Another study on six sea buckthorn varieties in Poland detected oxalic acid and isocitric acid (34).

Furthermore, sea buckthorn is rich in amino acids, which are indispensable to the human body. Amino acids are the basic units that make up proteins and are closely related to life activities. Seventeen amino acids, including seven essential amino acids (threonine, valine, methionine, isoleucine, leucine, phenylalanine, and lysine), have been detected in sea buckthorn fruits (39), leaves, branches and seeds (40). The amino acid content in sea buckthorn seeds is 18.63%, in leaves 15.41%, in branches 11.62%, and in fruits 6.89%. The content of aspartic acid and glutamic acid were highest in sea buckthorn fruits, leaves, and branches, with 1.11 and 1.24% in fruits, 2.42 and 1.60% in leaves, and 3.71 and 0.97% in branches. The highest content of tyrosine and glutamic acid can be found in sea buckthorn seeds, at 4.72 and 3.42%, respectively (40).

### Bioactive compounds

#### Carotenoids

Sea buckthorn fruits contain high levels of carotenoids, which give sea buckthorn its characteristic orange-yellow color. Carotenoids mainly act as antioxidants, although they also have other roles. For example, β-carotene is the precursor of vitamin A, and lutein/zeaxanthin constitutes the macular pigment of the eye (41). Carotenoids are considered to have health benefits and can reduce the risk of diseases, especially cancers and eye diseases (42). The content of carotenoids in different species and different parts of sea buckthorn varies greatly. Teleszko et al. (28) detected an average of 11 mg/100 g FW of total carotenoids in eight species of Russian sea buckthorn. In another study on six Romanian sea buckthorn varieties (*H. rhamnoides* ssp. *carpatica*), total carotenoid content ranged from 53 to 97 mg/100 g DW in berries, and ranged from 3.5 to 4.2 mg/100 g DW in leaves (18). β-Carotene is the main carotenoid in sea buckthorn. The percentage of β-carotene is 15–55% in berries, and 26–34% in the peel, pulp, and seed oil (28, 43). In addition, carotenoids include γ-carotene, *cis*-lycopene, lycopene, *cis*-γ-carotene, β-cryptoxanthin, α-carotene, and so on.

#### Polyphenols

Polyphenols are the main compounds with antioxidant activity in sea buckthorn. It has been reported that the polyphenol content in the fruit ranges from 12.36 to 34.6 mg GAE/g (GAE, gallic acid equivalents), higher than that in oranges (1.27 mg GAE/g) mandarins (1.16 mg GAE/g), blueberries (2.19 mg GAE/g), sour cherries (2.56 mg GAE/g), and strawberries (1.12 mg GAE/g) (29, 44, 45). A recent review showed that nearly 100 polyphenolic compounds have been isolated and identified from sea buckthorn (27). Polyphenols mainly include phenolic acids and flavonoids. Seventeen phenolic acids have been reported in sea buckthorn berries. Salicylic acid is the main phenolic acid in berries, accounting for 55–74.3% of the total phenolic acids (46). However, another study reported that gallic acid is the main phenolic acid in sea buckthorn fruit and leaves (27).

**FIGURE 2:**
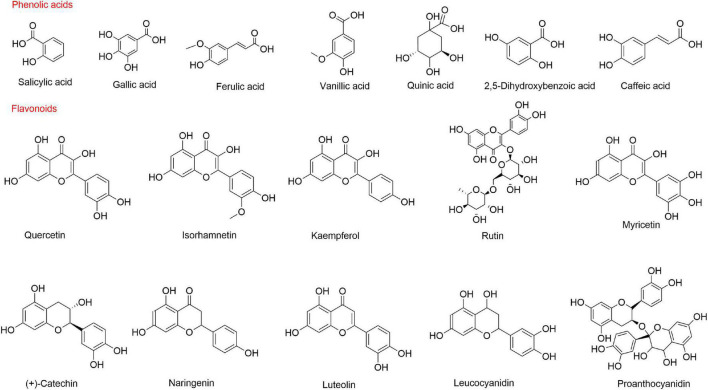
Structure of the main phenolic compounds in sea buckthorn.

Flavonoids may have potential roles in the prevention of chronic diseases, such as diabetes, cardiovascular disease, and cancer (47). Guo et al. (48) found that the total phenols and flavonoid aglycones in sea buckthorn extract had antioxidant and anti-proliferative activities. To date, 95 flavonoids have been identified from sea buckthorn, including 75 flavonols, 2 dihydroflavones, 6 catechins, 1 leucocyanidin, 9 anthocyanidins, 1 proanthocyanidin, and 1 chalcone (49). Raudonis et al. (50) detected the total flavonoid content in 11 sea buckthorn varieties grown in Lithuania and found that total flavonoid content ranged 385–616 μg/g FW. Flavonols are the major constituents of flavonoids and are mainly present in the glycosylated forms of quercetin, isorhamnetin, and kaempferol (49). Flavonols range from 463.14 mg to 893.92 mg/100 g DM, accounting for approximately 99% of the total phenolic compounds (34). The content and composition of polyphenolic compounds are significantly influenced by geographical factors, climatic conditions and berry varieties. Chemical structures of the main phenolic compounds in sea buckthorn are shown in Figure 2.

#### Fatty acids

Sea buckthorn is rich in a variety of fatty acids that play an important role in human health, such as treating skin and mucous membrane disorders and dry eyes syndrome and reducing the risk of cardiovascular disease (30). Teleszko et al. (28) identified 11 fatty acids in sea buckthorn pulp oil. At present, 24 fatty acids have been identified in wild and cultivated sea buckthorn berries in Lithuania (Table 3). There were differences in the content of fatty acids between wild and cultivated sea buckthorn. The monounsaturated fatty acid content of wild sea buckthorn berries was significantly higher than that of cultivated berries, while the levels of polyunsaturated and saturated fatty acids were higher in cultivated berries (31). The main fatty acids in sea buckthorn berries are palmitic, palmitoleic, and oleic acids.

#### Phytosterols

Phytosterols, as a bioactive component, can prevent cardiovascular diseases. A recent study found that the total phytosterol content of berry lipids from eight Russian sea buckthorn species ranged from 6168.24 to 13378.22 μg/100 mL. Fourteen sterol compounds have been detected in sea buckthorn pulp lipids, namely 4-desmethyl sterols (cholestanol derivatives, including β-sitosterol, stigmasterol, campesterol, and Δ^5^-avenasterol), 4α-monomethyl sterols (e.g., citrostadienol), and 4,4-dimethylsterols (e.g., 24-methylenecycloartanol) (Table 3) (28).

## Health benefits

Sea buckthorn contains a variety of bioactive components, including vitamins, carotenoids, polyphenols, fatty acids, and phytosterols. These components exert a wide range of health benefits by exerting antioxidant, anticancer, anti-inflammatory, antimicrobial and antiviral effects, as well as exerting protective cardiovascular, dermatological, neuroprotective, and hepatoprotective effects. The health benefits of sea buckthorn are categorized and summarized in Table 4, which highlights the study type, main results and potential bioactive components.

**TABLE 4:** Health benefits of sea buckthorn.

Sea buckthorn	Effective concentration/time	Study type	Experimental model	Main results	Bioactive compounds	References
**Antioxidant**						
Phenolic fraction from sea buckthorn fruits	0.5–50 μg/mL	*In vitro*	H_2_O_2_ or H_2_O_2_/Fe-treated human plasma or blood platelets	↓ Plasma lipid peroxidation and protein carbonylation. At 50 μg/mL, the inhibition rate of plasma lipid peroxidation was 60%	Flavonoids	(52)
Sea buckthorn extract	100 mg/kg⋅bw	*In vivo*	Hyperlipidemic rats	↓ Oxidative damage provoked by the lipid peroxidation	Polyphenols	(53)
Sea buckthorn leaf extracts	5, 10, 20 μg/mL	*In vitro*	PC-12 cells	↓ Relative proportion of total apoptotic PC-12 cells	Ellagic acid, gallic acid, isorhamnetin, kaempferol, and quercetin	(54)
Sea Buckthorn seed oil	500 ng/mL	*In vitro*	UV-Induced human skin cells	↓ ROS generation by approximately 25%	Fatty acids, phytosterols, vitamins A and E, β-carotene.	(55)
**Anticancer**						
Polyphenols extraction	80 and 120 μg/mL	*In vitro*	Human colon cancer cell	↓ Expression of cyclins and cell proliferation	Kaempferol and its derivatives	(56)
	50 mg/kg	*In vivo*	Xenograft BALB/c nude mice model	↓ Tumor volume and kinetic tumor growth		
Leaf aqueous extract	3.12, 6.25, 12.5, 25, 50 μg/mL	*In vitro*	LNCaP and C4-2 cell	↓ Proliferation and migration of prostate cancer cells	–	(12)
Leaf extract	6.2, 62 μg/mL	*In vitro*	Rat C6 glioma cells	↓ Intracellular ROS ↑ Pre-apoptosis in rat C6 glioma cells	Phenolics	(57)
Isorhamnetin	12.5, 15 μmoL/L	*In vitro*	Hypoxia model of CoCl_2_ (100 μmol/L) promoting maximal proliferation of MKN-45 cells	↓PI3K AKT mTOR-mediated adaptive autophagy ↑ MKN-45 gastric cancer cell apoptosis in a hypoxic environment	Isorhamnetin	(58)
**Anti-hyperlipidemia**						
Flavonoid-enriched extract from sea buckthorn seed	100 and 300 mg/kg	*In vivo*	High fat diet (HFD)-induced obese mouse model	↓ Serum and liver triglyceride concentrations in dose-dependent manner	Flavonoid	(64)
Sea buckthorn fruit oil	50, 100, 200 mg/kg	*In vivo*	Hypercholesterolemic golden syrian hamster model	↓ TC, TG, non-HDL-C ↑ HDL-C ↓ Oxidative stress and liver impairment caused by hyperlipemia through regulating antioxidant enzyme	Palmitoleic acid	(65)
**Anti-obesity**						
Sea buckthorn polysaccharide	0.1%	*In vivo*	High fat diet (HFD) induced C57BL/6 male mice	↓ Accumulation of lipids and weight gain	Polysaccharide	(66)
Sea buckthorn fruit oil	50, 100, 200 mg/kg	*In vivo*	Hypercholesterolemic golden syrian hamster model	↓ Weight and blood sugar elevation	Palmitoleic acid	(65)
Flavonoid-enriched extract from sea buckthorn seed	100 and 300 mg/kg	*In vivo*	High fat diet (HFD)-induced obese mouse model	↓ Serum and liver triglyceride concentrations in dose-dependent manner	Flavonoid	(64)
Sea buckthorn freeze-dried powder	4 mg/(g.d. body weight)	*In vivo*	High-fat induced obesity mice	↓ Body weight gain	–	(67)
**Antiplatelet**						
Polyphenols rich fraction from fruits	50 g/mL	*In vitro*	Healthy human blood platelet	↓ Platelet activation	Polyphenols	(68)
Non-polar fraction from twigs	10 μg/mL	*In vitro*	Healthy human blood platelet	↓ Platelet adhesion and platelet aggregation	Triterpenoids	(69)
Sea buckthorn fraction	0.5–50 μg/mL	*In vitro*	Human blood platelet	↓ Adhesion of resting platelets and thrombin-activated platelets to fibrinogen	Phenolic compounds	(70)
**Dermatological**						
Sea buckthorn extract	8 weeks (twice daily)	Clinical trial	10 psoriasis patients	↓ Psoriasis Area Severity Index (PASI) and Dermatology Life Quality Index (DLQI) scores	–	(71)
Sea buckthorn oil	100, 200 mg/kg p.o. 20 μL topical application (T.A.)	*In vivo*	TPA stimulated CD-1 mice psoriasis-like model	↓ Ear edema by 34.05 ± 7.65%, 30.45 ± 8.90%, respectively ↓Ear epidermal thickness by 31.80 ± 6.90 μm and 21.91 ± 5.07 μm, respectively,	Fatty acids	(72)
Sea buckthorn oil	1 mL/kg 4 weeks	*In vivo*	DNCB-induced AD-like lesions mice model	↓ DNCB-induced AD severity	–	(73)
Sea buckthorn cream	3-mm thickness (once a day)	Clinical trial	55 patients with second-degree burns	↓Period of wound healing and the course of treatment of second-degree burns	–	(74)
Sea Buckthorn seed oil	500 ng/mL	*In vitro*	UV-Induced human skin cells	↓ UV-induced disorders of redox and lipid metabolism in skin fibroblasts and keratinocytes	Fatty acids, phytosterols, vitamins A and E, β-carotene.	(55)
**Anti-inflammatory**						
Sea buckthorn peel extract	500 mg/kg	*In vivo*	48/80-induced rat paw edema models	↓ Edema volume	Ursolic acid, oleanolic acid	(75)
Sea buckthorn branches, berries, and leaves extracts	10 μg/mL	*In vitro*	RAW 264.7 macrophages	NO inhibition rates increased from 73 to 98%	Phenolic compounds	(76)
Sea buckthorn leaves extracts	0.05, 5, 50 μg/Ml	*In vivo*	Mouse peritoneal macrophages	↓ Pro-inflammatory cytokine level (TNF-α, IFN-γ and IL-6)	Tannins, proteins and carbohydrate groups	(15)
Sea buckthorn fruits powder	5, 10, 25, 50, and 100 μM	*In vitro*	RAW 264.7 (the mouse macrophage cell line)	↓ LPS-induced NO production	1,5-Dimethyl Citrate	(77)
Sea buckthorn flavonoids (Shanghai Yuan Ye Biotechnology, Ltd.)	0.06%, 0.31% w/w	*In vitro*	HFFD-induced obese mice	↓ Inflammatory mediators/cytokines, iNOS, COX-2, and IL-1β	Flavonoids	(78)
**Antimicrobial**						
Sea buckthorn leaf extracts	5%	*In vitro*	Common skin and wound pathogens	↓ Gram positive bacteria (*S. aureus, S. epidermidis*, *S. intermedius, and S. pyogenes*)	–	(16)
Sea buckthorn berries extract	0.15 mg/mL	*In vitro*	Human keratinocytes (HaCaT) cells	↓ Various proinflammatory cytokines and apoptotic pathways	–	(81)
Sea buckthorn berries and leaves extracts	6 mg/mL	*In vitro*	*Staphylococcus aureus* (MRSA)	Inhibits the growth of MRSA	–	(82)
**Antiviral**						
Sea buckthorn fruit peel extract	12.5 μM	*In vitro*	HSV-2 virus infected Vero cells	↓ Herpes simplex type 2 virus yield	14-Noreudesmanes and a phenylpropane heterodimer	(14)
Sea buckthorn leaf extract	100 mg/kg body weight	*In vivo*	Healthy Swiss albino mice	↑ RVNA titers and CTL population ↑ Memory T cells, plasma cells	Isorhamnetin and other flavonoids	(84)
**Neuroprotective**						
Sea buckthorn powder	1.5 g/mL	*In vitro*	Aβ-induced Neuroblastoma cells	↓ Intracellular Aβ depositions and Aβ-induced toxicity	–	(86)
Sea buckthorn flavonoids (Shanghai Yuan Ye Biotechnology, Ltd.)	0.06%, 0.31% w/w	*In vitro*	HFFD-induced obese mice	↓ Insulin resistance, neuroinflammation, and cognitive impairment in the CNS	Flavonoids	(80)
Sea buckthorn berries extract	mL/kg	*In vivo*	Iron-induced epileptic rats	↓ Memory impairment, anxiety-like behavior, histological impairments	–	(87)
**Hepatoprotective**						
Sea buckthorn flavonoids extracted by MCAE	200 mg/kg, po	*In vivo*	Tetracycline-induced ICR mice fatty liver model	↓ Liver Index, serum Index, TG, TC, LDL-C, AST, ALT	Flavonoids	(90)
Sea buckthorn fermentation liquid	1.75, 2.675, 5.35 g/kg	*In vivo*	Alcoholic liver disease mice model	↓ Kidney and spleen injury caused by alcohol, liver hypertrophy, and alcoholic fatty liver	Flavonoids, triterpenes and related SCFAs	(91)
Sea buckthorn berries extracts	20 and 40 mg/kg	*In vivo*	BDL-induced liver fibrosis model in rats	↓ liver fibrosis by inhibiting HSC activation	Flavonoids, phenolic acids	(92)
Sea buckthorn flavonoids from seeds	100 and 300 mg/kg	*In vivo*	High-fat diets-induced obese mice model	↓ Fat infiltration of liver tissues caused by high-fat diet and the expression of PPARγ in liver and white adipose tissues	Flavonoids	(93)

### Antioxidant activity

Many studies have confirmed the antioxidant activity of sea buckthorn *in vitro* and *in vivo*. Phenolic fraction from sea buckthorn fruits inhibits hydrogen peroxide (H_2_O_2_) or H_2_O_2_/Fe stimulated plasma lipid peroxidation and protein carbonylation. In fact, protein carbonylation is a relatively stable biomarker of oxidative stress. The phenolic constituents of sea buckthorn fruit reduced the concentration of carbonyl groups in plasma protein treated with H_2_O_2_ or H_2_O_2_/Fe. When plasma was treated with sea buckthorn phenolic fractions at a concentration of 50 g/mL for 60 min, the inhibition rate of plasma lipid peroxidation was as high as 60% (51). *In vitro* trials have shown that sea buckthorn extract with or without atorvastatin for the treatment of hyperlipidemia helped reduce the oxidative damage caused by lipid peroxidation (52). In addition, sea buckthorn leaf extract attenuates intracellular oxidative stress in a dose-dependent manner, thereby increasing neuronal PC-12 cell viability and membrane integrity (53). Serban et al. (54) retrieved 3,145 results from six databases, including PubMed, Scopus, Web of Science and others, among which 101 studies on cardiovascular disease showed that sea buckthorn fruit lowered blood cholesterol levels and reduced inflammation and oxidative stress parameters. Sea buckthorn seed oil inhibits ultraviolet (UV)-induced redox balance disturbance in skin cells. Gęgotek et al. (55) reported that fibroblast incubation with sea buckthorn oil causes decreases in reactive oxygen species (ROS) generation by approximately 25%. Sea buckthorn may be used as a natural source of antioxidants to prevent and treat diseases related to oxidative stress.

### Anticancer activity

At present, many studies have shown that the bioactive components in sea buckthorn have anticancer activity. Sea buckthorn polyphenols, the active ingredient of kaempferol and its derivatives, have shown significant anti-colon cancer activity *in vitro* and *in vivo*. Sea buckthorn polyphenols upregulate expression of microRNA (miR)-195-5p and miR-497-5p and down-regulate the expression of miR-1247-3p to suppress cyclins expression, thereby arresting the cell cycle in the G1 phase and affecting further proliferation of colon cancer. In addition, sea buckthorn polyphenols (50 mg/kg) significantly reduced tumor volume and control tumor growth in xenografted BALB/c nude mice *in vivo* (56). Sea buckthorn leaf aqueous extract effectively targeted androgen receptor (AR) and significantly downregulated androgen response genes, prostate specific antigen (PSA), eleven-nineteen lysine-rich leukemia 2 (ELL2), ELL-associated factor 2 (EAF2), calreticulin (CALR) *in vitro*. Sea buckthorn leaf aqueous extract can effectively inhibit proliferation and migration of prostate cancer cells. Therefore, sea buckthorn leaves hold promise as a functional food that may play a key role in the prevention of prostate cancer in high-risk populations. However, the potential bioactive compounds in sea buckthorn leaves are yet to be investigated for the development of new treatment options for prostate cancer (12).

Kim et al. (57) reported that sea buckthorn leaf extract at concentrations of 6.2 and 62 μg/mL significantly reduced the production of intracellular ROS by 16.3 and 42.3%, respectively, up-regulated expression of the pro-apoptotic protein B-cell lymphoma-2 (BCL2)-associated X (Bax) and inhibited the rapid proliferation of C6 glioma cells (11 and 49.5%). Therefore, sea buckthorn may be a potential source of pharmacological interventions for glioma treatment. In addition, isorhamnetin, the active component of sea buckthorn, increased expression of the mitochondrial pathway pro-apoptotic protein (cytochrome c-caspase 9-caspase 3) in gastric cancer cells in a hypoxic environment. It also significantly inhibited the autophagy of MKN-45 gastric cancer cells and promoted the apoptosis of gastric cancer cells by activating the Phosphoinositide 3-kinase (PI3K)-protein kinase B (AKT)-mammalian target of rapamycin (mTOR) signaling pathway (58).

In short, these studies support the anticancer effect of sea buckthorn and suggest that polyphenolic compounds may be responsible for its anticancer activity. The anticancer mechanisms of sea buckthorn are related to the expression of cyclin, proapoptotic proteins, autophagy of cancer cells, and related signaling pathways. However, there are few *in vivo* experiments and clinical trials on the anticancer effects of sea buckthorn. Thus, further research on the anticancer effects of sea buckthorn in humans is needed. A growing number of studies have found that carotenoids, especially lycopene, can reduce the risk of prostate, breast, lung, cervical and other cancers (59). However, there are almost no studies on the anticancer activity of sea buckthorn carotenoids. The anticancer activity of sea buckthorn carotenoid extracts is a promising research direction.

### Anti-hyperlipidemia activity

Hypercholesterolemia is an important risk factor for cardiovascular disease (60). The bioactive substance in the lipids of sea buckthorn pulp, phytosterols, plays an important role in the prevention of cardiovascular diseases, especially hypercholesterolemia (28). Numerous clinical trials have shown that spreads with added phytosterols have a stronger cholesterol-lowering effect, reducing low-density lipoprotein cholesterol (LDL-C) levels by about 10–15% (61). The mechanism of the hypocholesterolemic effect of phytosterols may be *via* the inhibition of endogenous cholesterol reabsorption and the promotion of its excretion in the form of neutral steroids (62). A meta-analysis from 11 independent randomized controlled trials concluded that supplementation with sea buckthorn berries/extracts significantly improved total cholesterol, triglyceride (TG), LDL-C, and high-density lipoprotein cholesterol (HDL-C) in subjects with hyperlipidemia, but not in healthy subjects (63).

*In vivo* animal trials showed that sea buckthorn has anti-hyperlipidemic effects. Flavonoid-enriched extract from sea buckthorn seed (FSH) at a dose of 100 and 300 mg/kg reduced serum and liver triglyceride concentrations by 16.67 and 49.56% in high fat diet (HFD)-induced obese mouse, respectively. FSH may improve lipid metabolism by inhibiting peroxisome proliferator-activated receptor gamma (PPARγ) expression, promoting PPARα expression, and suppressing adipose tissue inflammation (64). In addition, sea buckthorn fruit oil extract dose-dependently attenuated metabolic dysfunction in hamsters with hyperlipemia, including improving blood lipid composition (total cholesterol (TC), TG, HDL-C, and non-HDL-C levels), and relieving oxidative stress and liver impairment through the AMP-activated protein kinase (AMPK) and Akt pathways (65). In summary, sea buckthorn fruit, seed and oil are a source of phenolic compounds (especially flavonoids) and phytosterols. Sea buckthorn may be a valuable source of important bioactive compounds for the prevention and treatment of cardiovascular disease, which requires further research support.

### Anti-obesity activity

Sea buckthorn polysaccharide promotes expression of PPARγ-coactivator 1α (PGC1α), uncoupling protein-1 (UCP-1), and PR domain containing 16 (PRDM 16) in adipocytes to activate the brown adipocytes and improve thermogenesis, thus inhibiting the accumulation of lipids and weight gain (66). Palmitic acid-rich sea buckthorn fruit oil extract reduces the weight of hypercholesterolemic hamsters and blood sugar elevation caused by dyslipidemia. Therefore, sea buckthorn fruit oil can relieve obesity caused by hyperlipidemia (65). It has been reported that FSH at doses of 100 and 300 mg/kg significantly reduced body weight gain in HFD-induced obese mice by 33.06 and 43.51%, respectively (64). Sea buckthorn freeze-dried powder is made by low-temperature freeze-drying technology, allowing the powder to retain all of the plant’s useful nutrients and functional ingredients. Sea buckthorn powder improves HFD-induced obesity by altering the composition and structure of gut microbiome (67). Sea buckthorn is likely to develop into functional foods and dietary supplements for obese people.

### Antiplatelet activity

Anticoagulant and antiplatelet agents play an important role in the prevention and treatment of cardiovascular thrombotic events caused by various mechanisms. The polyphenols rich fraction of sea buckthorn fruit at the highest used concentration (50 μg/mL) has potent antiplatelet activity compared to polyphenol and triterpenic acid rich fractions from leaves and twigs. It has been shown to inhibit the expression of PAC-1 in three models of non-activated platelets, platelets activated by 10 μM adenosine diphosphate (ADP), and platelets activated by 10 μg/mL of collagen. This may be due to the inhibition of platelet aggregation as a result of the low expression of GPIIb/IIIa (68). Another report indicated that the non-polar fraction of sea buckthorn twigs showed stronger antiplatelet activity than the phenolic and non-polar fractions of leaves. This activity may be related to the regulation of arachidonic acid metabolism, changes in ROS concentration, and expression of platelet receptors (69). The 50 g/mL sea buckthorn fraction inhibited the adhesion of resting platelets and thrombin-activated platelets to fibrinogen by 65 and 55%, respectively (70).

### Dermatological effect

Sea buckthorn has been reported to have a wide range of dermatological effects. Clinical trials have demonstrated the anti-psoriasis effects of sea buckthorn. Boca et al. (71) treated 10 patients diagnosed with mild to moderate psoriasis with topical sea buckthorn fruit extract. When compared with placebo-treated patients, the Psoriasis Area Severity Index (PASI) score and Dermatology Life Quality Index (DLQI) scores in the treatment group were improved at both the fourth and eighth weeks of treatment. Sea buckthorn also exhibits anti-psoriatic and anti-atopic dermatitis activities in animal models. In the 12-*O*-tetradecanoylphorbol-13-acetate (TPA)-induced psoriasis-like lesion CD-1 mouse model, simultaneous oral (100 and 200 mg/kg) and topical (20 μL) application of sea buckthorn oil significantly inhibited ear edema (34.05 ± 7.65%, and 30.45 ± 8.90%, respectively) and reduced ear biopsy weights. Sea buckthorn oil has anti-inflammatory and anti-psoriatic properties. The possible mechanism for these effects may be that the high levels of fatty acids in sea buckthorn oil acts to inhibit reactive nitrogen and down-regulate nuclear factor kappa-B (NF-κB) protein and pro-inflammatory cytokines (72). Studies have suggested that 4 weeks of consecutive use of sea buckthorn oil decreases 2,4-dinitrochlorobenzene (DNCB)-induced atopic dermatitis (AD) severity in mice. This effect was due to inhibition of the thymus activation regulated chemokine (TARC) and macrophage-derived chemokine (MDC) in Interferon-γ (IFN-γ)/tumor necrosis factor-α (TNF-α)-stimulated HaCaT cells, which occurred by blocking activation of the NF-κB/signal transducerand activator of transcription 1 (STAT1) signaling pathway, thereby inhibiting the development of AD-like skin lesions. Sea buckthorn oil may be an effective therapeutic agent in the treatment of patients with AD (73).

Moreover, a randomized triple-blind clinical trial demonstrated that the healing period for second-degree burns in patients treated with 40% sea buckthorn cream was about 5 days shorter than for patients treated with 1% silver sulfadiazine dressings. The sea buckthorn cream had better clinical efficacy and shortened the healing time for second-degree burns (74). It was found that sea buckthorn seed oil promoted wound contraction by increasing hydroxyproline, hexosamine, DNA, and total protein content, which in turn promoted full-layer burn wound healing. The wound healing potential of sea buckthorn seed oil is dependent on the presence of omega-3 and omega-6 fatty acids, tocopherols and carotenoids (75). In addition, the palmitic acid-rich fraction purified from sea buckthorn seed oil has cell proliferation properties that promote growth of keratinocytes and dermal fibroblasts, which can be used to develop skin preparations and skin care products (76). UV light induces damage to the redox system and impairs lipid metabolism in skin fibroblasts and keratin-forming cells, and sea buckthorn oil can inhibit this effect and sea buckthorn seed oil may be a promising natural substance for skin photoprotection (55).

Overall, sea buckthorn has a therapeutic role in dermatology due to the high levels of saturated, monounsaturated and polyunsaturated fatty acids and other biological compounds that exert their effects. Determining the specific bioactive compounds and their mechanisms of action, however, requires further research.

### Anti-inflammatory activity

Sea buckthorn is widely used in traditional medicine to treat inflammatory diseases. The anti-inflammatory activity of sea buckthorn has been demonstrated in many *in vivo* studies. For example, 70% methanolic extract of sea buckthorn (500 mg/kg) inhibited 48/80-induced edematous inflammation and significantly reduced the volume of foot swelling in rats (0.660 ± 0.082 mL, compared to 0.935 ± 0.041 mL in the control). The anti-inflammatory effect of the peel extract was greatest (0.470 ± 0.124 mL, compared with 0.920 ± 0.111 mL for the control). Ursolic acid and oleanolic acid were the main active compounds in the peel extract. They may cause membrane stabilization by inhibiting mast cell degranulation (77). The anti-inflammatory activity of sea buckthorn branches, leaves, and fruits was measured by nitric oxide (NO) production, and it was found that treatment with 10 μg/mL sea buckthorn extracts inhibited NO by 73–98%. Cytotoxic effects of sea buckthorn have not been observed in 3-(4,5-dimethylthiazol-2-yl)-2,5-diphenyltetrazolium bromide (MTT) assays. Sea buckthorn extracts displayed good anti-inflammatory activities in RAW 264.7 macrophages (78). Sea buckthorn leaves extract exhibited potent anti-inflammatory activity against lipopolysaccharide (LPS) stimuli by inhibiting the expression of NO, inducible nitric oxide synthase (iNOS) and cyclooxygenase-2 (COX-2), and by decreasing levels of pro-inflammatory cytokines (15). Furthermore, sea buckthorn fruit extract, identified as a citric acid derivative, inhibited LPS-induced NO production in RAW 264.7 cells by inhibiting the expression of IκB kinase alpha/beta (IKKα/β), inhibitor of kappa Bα (I-κBα), NF-κB p65, iNOS, and COX-2, and the activities of interleukin 6 (IL-6) and TNF-α (79). Similarly, Mulati et al. (80) reported that sea buckthorn flavonoids significantly reversed high-fat and high-fructose diet (HFFD)-induced iNOS overexpression and reduced interleukin 1β (IL-1β) and COX-2 mRNA levels in the hippocampus of mice, suppressing the HFFD-induced inflammation reaction.

Therefore, the anti-inflammatory activity of sea buckthorn may be attributed to ursolic acid, oleanolic acid, citric acid derivatives and flavonoids. Its anti-inflammatory mechanism of action may be related to inhibition of the expression of pro-inflammatory cytokines (IL-6, IL-1β, and TNF-α) and a reduction in the production of pro-inflammatory mediators (NF-κB, iNOS, and COX-2). Sea buckthorn has shown promise as a source of bioactive compounds for the treatment of inflammatory diseases, but more *in vivo* and clinical studies are still needed to support this.

### Antimicrobial and antiviral activity

It has been reported that sea buckthorn exhibits antimicrobial activities *in vitro*. Verma et al. (16) found that sea buckthorn leaf extract was significantly effective against all 67 gram-positive bacteria recovered from clinical samples. Sea buckthorn leaf extract at a 5% concentration inhibited *S. aureus*, *S. epidermidis*, *S. intermedius*, and *S. pyogenes* growth by almost 50%. Sea buckthorn extract may reverse the detrimental effects of *S. aureus* on human keratinocytes by down-regulating various pro-inflammatory cytokines and apoptotic pathways, such as ILs, TNFs, transforming growth factors (TGFs), IFNs, fibroblast growth factors (FGFs), MAPKs, matrix metalloproteinases (MMPs), and caspases and Wnts molecular pathways (81). Additionally, a study showed that 6 mg/mL of sea buckthorn berry and leaf extract significantly inhibits the growth of Methicillin-resistant *S. aureus* (MRSA) (82). Smida et al. (83) reported that an experimentally designed mouthwash based on sea buckthorn pulp oil had bactericidal effects on some periodontal pathogens and had the ability to inhibit the formation of single-strain and multi-strain biofilms.

Moreover, sea buckthorn exhibits significant antiviral activity. 14-Noreudesmanes and a phenylpropane heterodimer isolated from the 70% methanol extract of sea buckthorn fruit inhibited the replication of herpes simplex type 2 (HSV-2) virus. Therefore, sea buckthorn may be a potential source of antiviral agents with anti-HSV-2 activity and may provide an alternative drug candidate for the treatment of patient populations infected with acyclovir- and penciclovir-resistant strains of HSV-2 (14). In addition, it has been shown that immunization with sea buckthorn leaf extract and inactivated rabies virus antigens (SBTE + Rb) increases rabies virus neutralizing antibody (RVNA) titers and the cytotoxic T lymphocytes (CTLs) response. Compared with the Rb immunized group, memory T cells and plasma cells in the SBTE + Rb immunized group were significantly increased by 5.5 and 1.9%, respectively. The components of sea buckthorn leaf extract that exert adjuvant activity may be isorhamnetin and other flavonoids (84).

### Neuroprotective activity

Alzheimer’s disease is a neurodegenerative disorder in which the typical histopathological changes are extracellular amyloid-β (Aβ) deposition and neurofibrillary tangles due to Tau protein hyperphosphorylation (85). Sea buckthorn removes intracellular Aβ deposits, with sea buckthorn powder at 1.5 g/mL being the most effective. This finding may be attributed to the higher levels of antioxidants present in sea buckthorn berry powder. Antioxidants inhibit Aβ-induced toxicity and prevent cell death by exerting a neuroprotective effect. Sea buckthorn holds promise as a potential therapeutic agent for the treatment of Alzheimer’s disease (86). Another study showed that sea buckthorn flavonoids stimulated insulin receptor substrate (IRS)/AKT activation, reduced protein tyrosine phosphatase 1B (PTP1B) expression and normalized insulin signaling pathways, neurogenic damage and ERK/CREB/BDNF signaling pathways. It inhibited insulin resistance and neuroinflammation, attenuated HFD-induced cognitive impairment, and effectively prevented memory loss (80). In addition, sea buckthorn improved epileptiform activity in the cerebral cortex and hippocampus in iron-induced epilepsy rats and reduced anxiety-like behavior, and improved memory impairment and histological damage in rats (87). In conclusion, sea buckthorn has been demonstrated to possess neuroprotective effects. The mechanisms include the removal of Aβ deposits, inhibition of Aβ-induced toxicity, and inhibition of insulin resistance and neuroinflammation. These effects may be attributed to the presence of flavonoids and other antioxidant compounds in sea buckthorn. In the future, it is necessary to investigate the neuroprotective effect of sea buckthorn on humans using clinical trials.

### Hepatoprotective activity

Sea buckthorn extract and sea buckthorn oil have significant hepatoprotective activities. Sea buckthorn oil is rich in carotenoids and may be an important source of bioavailable lutein (88). Carotenoids such as β-carotene, lycopene, lutein, and β-cryptoxanthin exhibit hepatoprotective activity by reducing oxidative stress and regulating lipid metabolism in hepatocytes (89). Biochemical and histopathological studies have shown that sea buckthorn flavonoid extract significantly improves biomarkers, including TG, TC, LDL-C, aspartate aminotransferase (AST), and alanine aminotransferase (ALT) in the serum and liver of non-alcoholic fatty liver mice. The therapeutic effects of sea buckthorn were superior to that of curcumin (90). Furthermore, sea buckthorn fruit fermentation solution regulates hepatic lipid metabolism and oxidative stress by modulating the composition of the intestinal microbiota. Thus, sea buckthorn prevents alcoholic liver disease and exerts hepatoprotective effect (91). Another study found that the active ingredients in sea buckthorn inhibited the activation of hepatic stellate cells, reduced inflammatory cytokine levels, and reduced the development of bile duct ligation (BDL)-induced fibrosis in rats in a dose-dependent manner. Thus, sea buckthorn reduces liver injury and inflammation, and restored liver function (92). It has been reported that a flavonoid extract from sea buckthorn seed residues reduces the number of adipocytes in the liver of obese mice, which significantly reduces HFD-induced fatty infiltration in the liver tissue, and reduces expression of PPARγ in the liver and adipose tissue, resulting in reduced of fat accumulation (93). In general, the flavonoids and carotenoids in sea buckthorn have hepatoprotective effects. The mechanisms for these effects may be associated with the regulation of lipid metabolism and oxidative stress and a reduction in inflammatory factor levels. It is necessary to conduct more *in vivo* studies to explore the hepatoprotective activity of sea buckthorn.

## Food applications

In addition to medical biological activities, sea buckthorn is also widely used in food, and has high economic value. Sea buckthorn is rich in nutritional value and contains a variety of biologically active compounds. Sea buckthorn is currently used as an antioxidant, antimicrobial and other natural additives in a variety of food products. The application of sea buckthorn in the food industry is more and more extensive, such as sea buckthorn oil, freeze-dried powder, fruit juice, fruit wine, milk tablets, fruit vinegar drinks, tea (94), preserved fruit, yogurt, and jam. The maximum utilization of sea buckthorn to improve the sensory properties and nutritional value of sea buckthorn products is currently being pursued by food industry manufacturers and researchers.

### Food additives

The meat processing industry is currently seeking natural additives to replace chemical additives in their products. Kozhakhiyeva et al. (95) found that the new functional cooked and smoked horse meat Jaya product, produced by adding 5.0% sea buckthorn fruit powder extract, is rich in 1.0% bioactive substances. The samples showed a 38% reduction in lipolysis and a significant 24% reduction in lipid hydroperoxides after 21 days of storage. This improved the oxidative stability and quality of new functional horsemeat delicacies. The addition of 3% ethanolic extract of sea buckthorn fruit to pork sausage effectively inhibited lipid oxidation and reduced the total bacterial count. Total sausage colonies were reduced by approximately 7 times, improving the microbiological content of the sausage (96).

The addition of sea buckthorn fruit powder to wheat bread extends the shelf life of the bread by 1–3 days. It also improves the antioxidant and organoleptic properties of the bread (8). The addition of 0.8 g/L of sea buckthorn leaf powder to white wine increased its free radical scavenging activity from 28.4 to 55.8%. The reducing ability of white wine, as measured by the amount of reduced ferric ion in an antioxidant power assay, increased from 35.3 to 62.1% with the addition of sea buckthorn. The total phenolic content of white wine increased from 11 to 23.7% and the color intensity increased from 39.9 to 50.7%, which contributed to the antioxidant capacity of the wines without sulfites (97). Sea buckthorn leaves have significant antioxidant capacity (98), and can be used as an alternative to increase the antioxidant capacity of wines.

Studies have found that sea buckthorn juice and its by-products could be used in chewing gum formulations and significantly improve the antioxidant activity. It showed antimicrobial properties against MRSA, *Klebsiella pneumoniae*, *Salmonella enterica*, *Pseudomonas aeruginosa*, *Bacillus cereus*, etc. Sea buckthorn juice and its by-products have great potential as antimicrobial agents in the food industry (99).

Furthermore, sea buckthorn seeds used to purify chitinase, *via* its action on the antifreeze protein HrCHI4 preserved the integrity of frozen green pea membranes and helped preserve sample freshness by retaining volatile compounds. This study opens up the possibility of using edible products to preserve food and preserve its texture and freshness by natural means (100).

All in all, sea buckthorn has a promising future as a natural food additive. The bioactive compounds contained in sea buckthorn, such as polyphenols (especially flavonoids), ascorbic acid, vitamins, carotenoids, and antifreeze proteins exert antioxidant, antibacterial and antifreeze effects. In the future, it will be necessary to investigate these mechanisms of action in depth for better application in food production.

### Sea buckthorn yogurt

Sea buckthorn, as a new plant-based additive, is becoming increasingly popular in dairy production worldwide due to its healthful, nutritional benefits. This is exactly the kind of nutritional quality that consumers are happy to seek. Sea buckthorn is rich in nutritional active substances and its addition to yogurt enhances the nutritional value of yogurt. Developed from sea buckthorn berries, sea buckthorn yogurt is rich in fat, protein, carbohydrates and antioxidants (vitamin C, vitamin E, carotenoids, phenols, etc.) meeting people’s nutritional needs. The yogurt can be stored safely at 4°C for 12 days and at 15°C for 3 days without losing its microbiological quality (9). In addition to adding sea buckthorn to yogurt, carrot (101), tomato (102), water chestnut (103), yellow peach, and passion fruit have also been added to sea buckthorn yogurt to develop novel healthy yogurt. The different additions add to the unique natural flavor of fruits and vegetables, enriching the yogurt with a variety of functional ingredients and making up for the nutritional deficiencies of plain yogurt (101).

### Sea buckthorn jam and jelly

Sea buckthorn berries have a sour taste and a short shelf life. Therefore, processing berries into jam is an effective means to improve sensory characteristics and increase berry utilization. The jam, produced using sea buckthorn fruit at 102°C with stevia, contains high levels of total carotenoids and polyphenols and exhibits antioxidant activity. After 21 days of storage at room temperature, the value of yeast and mold was less than 100 CFU/g, and the value of Enterobacteriaceae was less than 5 CFU/g (104). Ordinary jam has a single flavor. In the *Elaeagnus angustifolia* and sea buckthorn compound jam, sea buckthorn was used both as a raw material and as an acidulant instead of citric acid. The jam has a shelf life of 177 days at 20°C without the addition of preservatives (105). In addition, sea buckthorn can be combined with sweet potatoes, pumpkins and carrots in a certain ratio to make novel, nutritious and healthy compound jam (106). A reasonable mixture of sea buckthorn juice with other fruit juices (papaya, watermelon, grape) can produce a delicious and nutritious jelly. Among them, sea buckthorn mixed jelly prepared in certain ratios with grapes has shown good organoleptic characteristics. The shelf life of sea buckthorn-grape jelly is 6 months at room temperature and its microbial load is also within the specified limits (107). Sea buckthorn has the potential to be a potentially rich source of bioactive compounds for the production of sugar-based products.

### Sea buckthorn beverages

Sea buckthorn berry wastes are inevitably generated in the sea buckthorn processing industry, and improper disposal of these wastes will cause environmental pollution. Fermentation and reuse of these wastes can improve the utilization rate of sea buckthorn and increase the economic value of these wastes. Waste from the sea buckthorn processing industry can be used as a suitable substrate for fermentation. Fermentation under optimal fermentation conditions resulted in 3% ethanolic sea buckthorn beverage. This beverage contains high levels of phenolic compounds (including gallic acid, protocatechuic acid, vanillic acid, chlorogenic acid, etc.) and high antioxidant activity, and contains carbon dioxide and low levels of ethanol. So it is a refreshing and healthy functional drink (108). In addition to fermentation, a waste-free whole fruit pulp juice of sea buckthorn can be developed using the micro-wet milling (MWM) process. Compared to mixed milled and commercial sea buckthorn juice, MWM sea buckthorn juice has a better color (bright yellow tint, highest total carotenoid content of 145 ± 0.10 mg/mL), smaller particle size, higher ascorbic acid value (67.67 ± 1.15 mg/mL), total phenolic content and antioxidant activity. The process minimizes the loss of heat-sensitive bioactive compounds. It also provides a fiber-rich juice, showing great promise for processing sea buckthorn juice in the food industry (109). Sea buckthorn is rich in nutrients and bioactive compounds. The potential of sea buckthorn as a botanical ingredient for novel functional food applications is obvious. It is promising to make full use of sea buckthorn fruits, peels, and seeds and to explore new ways of processing sea buckthorn.

## Toxicity and safety

Despite the long nutritional and medicinal history of sea buckthorn, there is still relatively little information available on the toxicity and safety of sea buckthorn. Wen et al. (110) reported that sea buckthorn berry oil was not a genotoxic or teratogenic substance. He found that sea buckthorn berry oil showed no mutagenic activity against histidine-dependent strains of *Salmonella typhimurium* at exposure concentrations ranging from 8 to 5,000 μg/plate. At a dose up to 9.36 g/kg body weight, sea buckthorn berry oil had no significant effect on sperm morphology and the micronucleus rate of polychromatic erythrocytes in mice. In addition, 4.68 g/kg of sea buckthorn fruit oil did not cause maternal toxicity or embryotoxicity in pregnant mice. The no-observed-adverse-effect level (NOAEL) for rats was determined to be 4.68 g/kg of body weight. A 90-day safety study showed that the NOAEL in rats was 100 mg/kg body weight/day of aqueous fruit extract of sea buckthorn (111). Furthermore, Zhao et al. (112) reported that the maximum tolerated dose of sea buckthorn oil in the acute toxicity study in mice was bigger than 18.72 g/kg. Ninety-day repeated oral toxicity tests in rats showed that the NOAEL was 9.36 g/kg body weight. Sea buckthorn is healthy and non-toxic enough to be used as a human food, medicinal product, or dietary supplement.

## Conclusion and outlooks

Sea buckthorn is a unique and highly valuable plant with a wide distribution covering more than 2 million hectares of land worldwide. It contains nearly 200 bioactive compounds and has great benefits to human health, and *in vivo*, *in vitro*, and clinical trials in the past 5 years have demonstrated the health benefits of sea buckthorn. These biological activities include antioxidant, anticancer, anti-inflammatory, anti-hyperlipidemic, anti-obesity, antimicrobial and antiviral, dermatological, neuroprotective, and hepatoprotective effects. Moreover, sea buckthorn has created enormous economic value in the food industry and is a promising dietary source of bioactive components.

As a versatile economic and ecological plant, sea buckthorn undoubtedly has a bright future. This ancient plant has powerful therapeutic synergies and has made many contributions to humankind. Sea buckthorn has an outstanding ability to help with economic development and improve the ecological environment. In order to rationally develop and utilize sea buckthorn resources, further research should focus on: (1) Developing mechanical harvesting and reasonable preservation technology. Sea buckthorn is a berry plant and manual harvesting is inefficient. The berries have high moisture content and are easily squeezed leading to deformation and mold. It is necessary to improve the efficiency at the harvesting stage; (2) Isolation and identification of more specific bioactive compounds and further study of their health promoting mechanisms; (3) Conducting more clinical trials to verify the health benefits of sea buckthorn for humans; (4) Applying sea buckthorn in the prevention and treatment of diseases; (5) Developing functional foods and other products based on sea buckthorn; (6) Adhering to the mode of combining economy and ecology, and developing the sea buckthorn industry in a scientific, reasonable and sustainable manner.

## Author contributions

QM conceptualized the topic. FZ and PW reviewed the literature and collected the data. ZW drafted the manuscript. GH and XC revised and edited the manuscript. All authors contributed to the article and approved the submitted version.
